# Neuropathic Corneal Pain after Coronavirus Disease 2019 (COVID-19) Infection

**DOI:** 10.3390/diseases12020037

**Published:** 2024-02-09

**Authors:** Natalie Shi Qi Wong, Chang Liu, Molly Tzu-Yu Lin, Isabelle Xin Yu Lee, Louis Tong, Yu-Chi Liu

**Affiliations:** 1 Yong Loo Lin School of Medicine, National University of Singapore, Singapore 117597, Singapore; 2 Singapore Eye Research Institute, Singapore 169856, Singapore; chang.liu@seri.com.sg (C.L.);; 3 Department of Cornea and External Eye Disease, Singapore National Eye Centre, Singapore 168751, Singapore; 4 Ophthalmology and Visual Sciences Academic Clinical Program, Duke-NUS Medical School, Singapore 169857, Singapore

**Keywords:** neuropathic corneal pain, coronavirus disease 2019, corneal nerves, cornea

## Abstract

Introduction: This is a case report of a patient with neuropathic corneal pain after coronavirus disease 2019 (COVID-19) infection. Methods: A previously healthy 27-year-old female presented with bilateral eye pain accompanied by increased light sensitivity 5 months after COVID-19 infection. She was diagnosed with neuropathic corneal pain based on clear corneas without fluorescein staining, alongside the presence of microneuromas, dendritic cells, and activated stromal keratocytes identified bilaterally on in vivo confocal microscopy. Results: The patient’s tear nerve growth factor, substance P, and calcitonin gene-related peptide levels were 5.9 pg/mL, 2978.7 pg/mL, and 1.1 ng/mL, respectively, for the right eye and 23.1 pg/mL, 4798.7 pg/mL, and 1.2 ng/mL, respectively, for the left eye, suggesting corneal neuroinflammatory status. After 6 weeks of topical 0.1% flurometholone treatment, decreased microneuroma size, less extensive dendritic cells, and reduced tear nerve growth factor and substance P levels were observed. The scores on the Ocular Pain Assessment Survey showed an improvement in burning sensation and light sensitivity, decreasing from 80% and 70% to 50% for both. Conclusions: Neuropathic corneal pain is a potential post-COVID-19 complication that warrants ophthalmologists’ and neurologists’ attention.

## 1. Introduction

Neuropathic pain, according to the International Association for the Study of Pain (IASP), is defined as “pain caused by a lesion or disease of the somatosensory nervous system” [1]. In the cornea, this is termed neuropathic corneal pain (NCP). Patients with NCP describe their corneal pain as “dry”, “burning”, hyperalgesia (disproportionate pain from a stimulus), or a “foreign body sensation”, which are often aggravated by light (photoallodynia), extremes of temperature, and dry wind, with or without associated symptoms such as tearing or itchiness [2]. This condition may result in disturbances in vision and blurring, significantly affecting quality of life [3,4]. Most commonly, NCP is caused by chronic dry eye disease but can be iatrogenic after refractive or cataract surgery [5,6] or result from various corneal pathologies, such as infectious keratitis, chronic contact lens wearing, or chemical injury [7]. Other causes include direct corneal nerve involvement, such as diabetes corneal neuropathy [8] and herpes zoster [9], as well as autoimmune conditions such as Sjogren’s syndrome [10]. The diagnosis of NCP is challenging due to the poor understanding of NCP as well as the lack of specific clinical signs [11]. The diagnostic workup includes the clinical assessment of pain-related symptoms, minimal or no corneal fluorescein staining on slit lamp examination, and corneal nerve abnormalities [11]. In vivo confocal microscopy (IVCM) has been used to reliably evaluate corneal nerve status and cellular morphology in ophthalmology [12], and scans of patients with NCP typically show structural nerve changes such as decreased nerve density or length and the presence of neuromas [13]. After an insult to the corneal nerves, subsequent nerve regeneration may result in neuroma formation, which often has dysfunctional responsiveness and spontaneous discharges, causing chronic ocular pain [14]. Hence, neuroma formation has also been described as a hallmark of neuropathic pain [15,16].

The recent coronavirus disease 2019 (COVID-19) pandemic resulted in the advent of research on various systemic complications after COVID-19 infection, including neuropathic pain in approximately 2% of patients, which may also occur in the richly innervated cornea [17,18]. Peripheral neuropathic pain in post-COVID-19 patients has been found in tandem with prolonged myalgia [19], and it can also manifest as trigeminal neuralgia [20] or even Guillain–Barré syndrome [21]. Bitirgen et al. also reported post-COVID-19 patients with neurological symptoms and significantly reduced corneal nerve fibre length (CNFL) as well as lower corneal nerve fibre density (CNFD) and corneal nerve branch density (CNBD) 3 months after COVID-19 diagnosis [22]. Furthermore, several review papers have reported that neurological sequelae, such as peripheral neuropathy and neuropsychiatric disorders, could occur months after COVID-19 infection [23].

There have been a few small studies reporting morphological changes in the corneal nerve, such as CNFD, CNFL, and CNBD, as well as increased neuroma and dendritic cell formation observed on IVCM in post-COVID-19 patients compared to healthy controls [24,25]. In addition, altered levels of neuromediators, such as nerve growth factor (NGF), substance P, and calcitonin gene-related peptide (CGRP), have been found in COVID-19 patients [26]. Studies have shown increased serum and salivary NGF levels in the acute phase in COVID-infected patients, suggesting its use as an early biomarker of morbidity [27,28]. A few reports have also described higher serum levels of CGRP in acute COVID-19 infection and have proposed CGRP antagonists as a novel therapeutic agent for COVID-19 [29,30]. These neuromediators also play an important role in maintaining corneal nerve health and ocular surface homeostasis [31]. However, the changes in neuromediators specific to patients with NCP or any neuropathic pain before and after COVID-19 infection have not yet been investigated.

This report presents a case with NCP possibly secondary to COVID-19 infection. We herein reported detailed subjective and objective ocular surface assessment and imaging of morphological changes in the corneal nerve plexus, epithelial and stromal cells, and tear neuromediator profiles. Changes found via the above-mentioned evaluation after topical corticosteroid treatment are also documented.

## 2. Detailed Case Description

**Figure 1:**
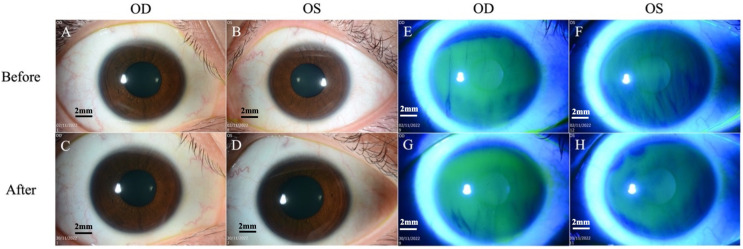
Slit lamp biomicroscopy images. (**A**,**B**) Cornea is clear on both eyes on slit lamp examination before treatment. (**C**,**D**) Cornea remains clear after treatment. (**E**,**F**) No fluorescein staining of the cornea and conjunctiva before treatment. (**G**,**H**) No fluorescein staining after treatment. Scale bar: 2 mm.

**Figure 2:**
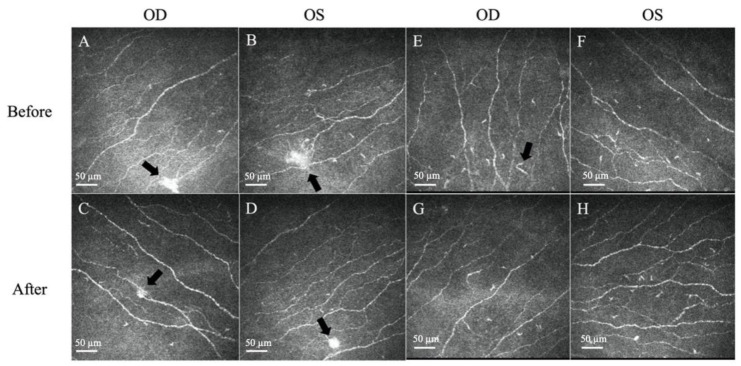
Representative in vivo confocal microscopy (IVCM) images of corneal neuromas and dendritic cells. (**A**,**B**) The presence of neuromas before treatment, manifesting as irregularly shaped, hyper-reflective enlargements of terminal nerve endings (arrows). (**C**,**D**) The size, area, and perimeter of the neuromas decreased after treatment with topical steroids (arrows). (**E**,**F**) The presence of dendritic cells, which appear as bright hyper-reflective cell bodies before treatment. (**G**,**H**) The dendritic cell count and area slightly decreased after treatment. Scale bar: 50 µm.

**Figure 3:**
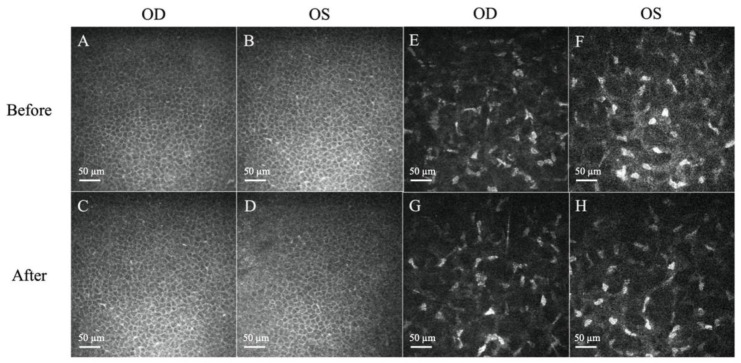
Representative in vivo confocal microscopy (IVCM) images of corneal epithelium and stroma. (**A**–**D**) Normal, regularly shaped cells with bright cell borders and dark cytoplasm were observed before and after treatment. (**E**,**F**) Presence of hyper-reflectivity of keratocytes and prominent cytoplasmic extensions before treatment. (**G**,**H**) The reflectivity of the stroma decreased after treatment. Scale bar: 50 µm.

A 27-year-old female, previously healthy with no history of ocular surgery or ocular diseases, presented to the emergency department with bilateral eye pain, with the right eye worse than the left. The ocular pain started after her COVID-19 infection (confirmed by polymerase chain reaction test) 5 months prior and had been worsening and becoming intolerable. She denied any personal medical history of diabetes mellitus, anxiety, depression, fibromyalgia, irritable bowel syndrome, autoimmune disease, or any other potentially associated conditions, and was fully vaccinated for COVID-19 9 months prior to her COVID-19 infection. An initial impression of dry eye disease was made, and the patient was discharged with topical lubricants. As her pain symptoms had not subsided, she sought a second opinion. Her visual acuity was 6/6 in both eyes, and the fundus examination was unremarkable. Slit lamp examination revealed a clear cornea with no corneal fluorescein staining for either eye (Figure 1). The lid margin and meibomian gland openings were normal. There were no signs of conjunctivochalasis or allergic conjunctivitis, such as conjunctival congestion, papillary reaction, or chemosis. The tear break-up time was 4 s, and the Schirmer I test was 5 mm bilaterally. She was continued on topical lubricants, and ciclosporin eyedrops (Ikervis^®^, Santen Pharmaceuticals, Osaka, Japan) were added. However, the pain persisted, with worsening photosensitivity bilaterally. As the pain was disproportionate to the clinical ocular surface assessment, an IVCM scan was performed to evaluate her corneal nerve status. On IVCM images, epithelial cells with normal bright cell borders and dark cytoplasm with regular shapes were observed. Corneal sub-basal nerves were clearly visible, with the presence of neuromas, which appeared as irregularly shaped, hyper-reflective enlargements of terminal nerve endings. Some corneal dendritic cells were also observed in the sub-basal and subepithelial layers. The stromal keratocytes were hyper-reflective and had prominent cytoplasmic extensions in the stroma (Figure 2 and Figure 3).

Corneal nerves were further analysed and quantified by ACCMetrics (University of Manchester, Manchester, UK) [32,33]. Corneal epithelium, neuromas, and dendritic cells were analysed using the AIConfocal Rapid Image Evaluation System (ARIES; ADICS, Saint-Contest, France) [34]. The detailed results are provided in Table 1.

Her corneal sensitivity, measured with a Cochet-Bonnet esthesiometer, was 30 cm for both eyes (0–6 cm for each area, 0–30 cm for the central area and four quadrant areas) [35]. In the proparacaine challenge test, the pain score (range 0–10) was reduced from 5 and 1 in her right and left eyes, respectively, to 0 bilaterally after application of 0.5% topical Alcaine (Alcon, Geneva, Switzerland). The abolishment of pain after administration of proparacaine suggests peripheral NCP rather than central NCP [11]. In the evaluation of symptom severity using the Ocular Pain Assessment Survey (OPAS) questionnaire, the scores ranged from 70% to 90% for all items. Of significance, she scored 80% for burning sensation and 70% for sensitivity to light (Table 2). Tear samples were analysed using enzyme-linked immunosorbent assay (ELISA) with the protocol published previously in [36]. In brief, thin Schirmer strips with tear fluid samples were cut; submerged in 200 µL elution buffer consisting of 0.55 M NaCl, 0.33% Tween-20, 0.55% bovine serum albumin, and protease inhibitor; and then subjected to agitation and sonication at 450 rpm for 17 h at 4 °C. The eluted tear proteins were subsequently centrifuged, and the clear supernatants were collected. ELISAs were then performed according to the manufacturer’s protocol: substance P (6× dilution), CGRP (4× dilution), and NGF (1.5× dilution), respectively (CGRP from Phoenix Pharmaceuticals, Runcorn, UK; Substance P and NGF from R&D Systems, Minneapolis, MN, USA). Tear samples were subjected to analysis using enzyme-linked immunosorbent assay (ELISA): substance P (6× dilution), CGRP (4× dilution), and NGF (1.5× dilution), respectively (CGRP from Phoenix Pharmaceuticals, Runcorn, UK; substance P and NGF from R&D Systems, Minneapolis, MN, USA). The levels of tear NGF, substance P, and CGRP were 5.9 pg/mL, 2978.7 pg/mL, and 1.1 ng/mL, respectively, for the right eye and 23.1 pg/mL, 4798.7 pg/mL, and 1.2 ng/mL, respectively, for the left eye.

Topical 0.1% fluorometholone eyedrops, twice daily, were prescribed. After 6 weeks of topical fluorometholone treatment, the patient’s subjective symptoms improved to a score of 50% for both burning sensation and sensitivity to light (Table 2). The average neuroma area, perimeter, and size, as well as the dendritic cell counts and density, were reduced bilaterally (Figure 2 and Figure 3, Table 1). The levels of the tear neuromediators NGF, substance P, and CGRP largely decreased after treatment to 3.2 pg/mL, 3114.4 pg/mL, and 0.8 ng/mL, respectively, for the right eye and 0.1 pg/mL, 2782.2 pg/mL, and 1.1 ng/mL, respectively, for the left eye.

## 3. Discussion

While the pathophysiology of how the SARS-CoV-2 virus impacts peripheral nerves is still unknown, Shiers et al. reported that human primary sensory neurons express the angiotensin-converting enzyme 2 (ACE2) receptor, which is commonly found in SARS-CoV-2 [37]. This suggests that this receptor possibly serves as the entrance pathway of the virus into ocular nociceptor neurons, which is the first step in the generation of pain [37,38]. Parallel infection of corneal epithelial cells through the same mechanism may cause an inflammatory response that contributes to the damage of corneal nerve terminals, microneuromas, and abnormal expression of ion channels in pain nerve fibres, leading to aberrant nerve activity that causes neuropathic pain [39,40].

The IVCM findings of the presence of neuromas, increased dendritic cell counts, and activated stromal keratocytes corroborated the morphological changes found in NCP in the literature, which form in response to nerve injury and inflammation [13]. Corneal neuromas, described as enlarged terminal nerve endings, are a result of healing attempts directed by the surviving Schwann cell tube in response to axonal injury and are highly specific for neuropathic corneal pain [41,42]. Cytokines and growth factors secreted in response to nerve injury and inflammation also activate the usual quiescent keratocytes, gaining replicative, migratory, and contractile properties, which cumulatively manifest as hyper-reflective keratocytes [42,43]. Dendritic cells are the most potent antigen-presenting cells in the body, and increased mature dendritic cells are seen in active corneal inflammation [44,45]. Barros et al. reported that dendritic cells were found in corneas in almost 70% of post-COVID patients, with a predilection for younger, asymptomatic patients [25]. It was also found that several receptors, such as CD209, CD26, CD30, and CD66, found on the SARS-CoV-2 virus were also expressed in dendritic cells, explaining their presence in even asymptomatic COVID-19 patients [46,47].

Several IVCM studies have presented corneal nerve changes after COVID-19 infection. However, in those reports, the nerve changes did not manifest as NCP. Mirza et al. and Bitirgen et al. reported significantly reduced CNFL, CNFD, and CNBD in post-COVID-19 patients with neurological symptoms, which persisted after 3 months post-COVID-19 diagnosis, when compared to asymptomatic post-COVID-19 patients and healthy controls [22,24]. This may explain why, aside from the well-documented anosmia and dysgeusia [48], there have also been various neurological manifestations, such as ophthalmoplegia, Millie Fisher syndrome, and trigeminal neuropathy, reported in COVID-19 patients [49,50]. Another study found neuroma-like structures, nerve beading, and the presence of abundant dendritic cells in 21 out of 23 post-COVID-19 patients [25], which was consistent with the findings in this case. In this case, a significant reduction in CNFL and CNFD was not observed compared to those published values analysed with the same software [51,52]. This might be because the IVCM scan was performed in a relatively early stage in the post-COVID-19 period compared with other studies [32,38]. This also highlights the fact that neuropathic pain symptoms can occur in the absence of marked nerve reduction. In fact, nerves with neuromas are hyperexcitable and can elicit spontaneous and ectopic discharge, causing pain and pain-like symptoms such as hyperalgesia, a burning sensation, or allodynia [41,53]. At present, only one case of NCP, which was accompanied by headache symptoms, after long-COVID-19 infection has been reported [54]. It was treated as centralized NCP because topical oxybuprocain failed to provide an analgesic effect, and systemic medications such as duloxetine and carbamazepine were administered for neuropathic pain. In contrast, our patient had peripheral NCP and responded well to topical corticosteroid treatment. Tricyclic antidepressants inhibit serotonin and norepinephrine reuptake and block cholinergic, histaminergic, and sodium channels [55], while anticonvulsant carbamazepine blocks sodium channels [56]. Calcium channel α 2-δ ligands (Gabapentin and pregabalin) bind to α2-δ subunit voltage-gated calcium channels and inhibit glutamate, norepinephrine, and substance P release, stabilizing neurons [11,57]. All these drugs have been shown to be effective in the management of NCP [58,59,60]. An opioid antagonist has been suggested to reduce pain and cytokine release in NCP [61,62,63]. Topical administration of opioids might be more effective at avoiding potential side effects. A few studies have revealed that topical opioids reduced pain scores and increased rate of epithelial healing post-photorefractive keratectomy without deleterious effects [64,65]. A phase I study indicated the tolerability to escalating doses of topical naltrexone (1–4 eye drops at dosages up to 50 μM) in healthy individuals [66].

We investigated the concentrations of NGF, substance P, and CGRP, as they are common neuromediators that are involved in ocular surface neuroinflammation [67]. Compared to the published concentrations of neuromediators in tears (substance P: 1926.3–2672.0 pg/mL; NGF: 5.4–10.0 pg/mL; CGRP: 0.9–2.6 ng/mL) [36,51,68], increased tear NGF and substance P levels were found in this case, which were suppressed after topical corticosteroid treatment. These neuromediators are released in response to neuroinflammation, the pathophysiological process that underlies NCP. Increased tear neuromediators have also been described in chronic pain. For example, substance P has been shown to be upregulated in the lumbar spinal cord of rat models with neuropathic pain [69], anti-NGF antibodies have been found to alleviate chronic neuropathic pain in rat models [70], and CGRP monoclonal antibodies have been reported as novel biologics for the treatment of migraines and cluster headaches [71]. Blood-derived eye drops, such as autologous serum tears (ASTs), have been shown to be effective in alleviating pain and promoting nerve regeneration in NCP. However, the limitations of blood derivates are the limited availability, high cost, and the storage requirements [13,41]. Topical corticosteroids are a mainstay of treatment for peripheral NCP, especially for acute pain relief [2]. They exert anti-inflammatory (including anti-neuroinflammatory) effects and hence analgesic effects by inhibiting cellular infiltration, capillary dilation, and fibroblast proliferation [72] by inhibiting inflammatory cascades and proinflammatory molecule synthesis. Topical corticosteroids also help reverse nerve abnormalities such as nerve thickening and tortuosity in NCP [73]. This case study found that corneal dendritic cells decreased after topical corticosteroid treatment, which is in line with previous reports that showed decreased dendritic cell density after topical corticosteroid treatment compared to baseline in dry eye disease [74]. NCP remains an ill-defined entity, and the diagnosis of NCP requires the exclusion of ocular comorbidities. Several ocular surface assessments, such as meibography or tear osmolarity, which were not performed in this case study, can be considered to rule out possible comorbidities. Nonobvious obstructive meibomian gland dysfunction, in which the meibomian glands could be normal and the eyelid margin could be without inflammation [75], should be ruled out. Moreover, a larger cohort would be required to better understand the underlying pathogenesis and disease course.

## 4. Conclusions

In conclusion, this case report presents for the first time the clinical manifestations, nerve imaging features, and neuromediator profiles of a case of NCP after COVID-19 infection before and after treatment. NCP can be a potential post-COVID-19 complication that warrants ophthalmologists’ and neurologists’ attention, as it poses a diagnostic challenge and is potentially debilitating to patients’ quality of life.

## Figures and Tables

**Table 1:** Analysis of corneal nerves, corneal neuromas, and dendritic cells before and after treatment.

	Before		After	
Parameter	OD	OS	OD	OS
Neuroma parameters				
Total area (µm^2^)	462.85	751.56	478.54	398.96
Average size (µm)	126.33	38.79	86.43	33.25
Perimeter (µm)	37.73	23.69	37.75	22.58
Dendritic cell parameters				
Count	22.4	20.4	18.67	11.00
Density (/µm^2^)	0.022	0.022	0.023	0.022
Area (µm^2^)	45.27	46.39	43.48	45.46
Nerve parameters				
CNFD (number/mm^2^)	19.53	19.14	24.34	21.71
CNBD (number/mm^2^)	23.44	21.87	21.71	22.83
CNFL (mm/mm^2^)	12.40	12.82	13.06	12.83
CTBD (number/mm^2^)	36.72	37.50	30.26	35.41
CNFA (mm^2^/mm^2^)	0.0057	0.0069	0.0058	0.0058
CNFW (mm/mm^2^)	0.021	0.021	0.022	0.020
Cfradim	1.46	1.46	1.47	1.46

CNFD = corneal nerve fibre density; CNBD = corneal nerve branch density; CNFL = corneal nerve fibre length; CTBD = corneal nerve fibre total branch density; CNFA = corneal nerve fibre area; Cfradim = corneal nerve fractal dimension.

**Table 2:** Truncated Ocular Pain Assessment Survey (OPAS) responses before and after treatment.

	Before	After
Aggravating factors—% increase in pain when exposed to:		
Wind, dry air, heat, air conditioning	80%	60%
Volatile chemicals (cleaning agents, fumes, cosmetic fragrances)	90%	80%
Associated symptoms—% frequency that ocular pain is accompanied by:		
Redness	70%	50%
Burning	80%	50%
Sensitivity to light	70%	50%
Tearing	80%	80%

## Data Availability

All data generated or analysed during this study are included in this published article.
